# Dynamic conflict among heterogeneous groups: a comment on Christensen and Radford

**DOI:** 10.1093/beheco/ary044

**Published:** 2018-04-27

**Authors:** Faye J Thompson, Michael A Cant

**Affiliations:** Centre for Ecology and Conservation, University of Exeter, Penryn Campus, Penryn, Cornwall, UK

Christensen and Radford (2018) provide a stimulating review of one of many poorly understood aspects of intergroup conflict, “neighbour stranger response differences” or NSRD. We applaud Christensen and Radford for drawing together a disparate literature on group-living species, and for insightful discussion of the complexities of intergroup interactions. NSRD has been the topic of much research in nonsocial species, so it seems natural to ask whether this research helps to understand variation in conflict behavior between heterogeneous groups composed of individuals with varying interests. Sometimes, however, we believe that research on intergroup conflict can be obscured rather than clarified by theory and hypotheses derived to explain individual-level conflict and territoriality.

In this case, we think that the focus on NSRD distracts from the most important and interesting feature of intergroup conflict: the intrinsic variability and dynamics of the parties involved. As Christensen and Radford’s review comprehensively demonstrates, neighbors and strangers alike can vary hugely in the threat they pose, depending on reproductive status (e.g. whether females are receptive to mating, or caring for young offspring), group size and composition (e.g. the number of individuals that are predominantly involved in fighting), and their motivation for interacting with a rival group (e.g. to gain territory, or compete for food or mates). Similarly, the threat to own group (and therefore the likely response to intergroup conflict) can also vary depending on own group traits. This variation within and between the parties involved in intergroup conflict is likely to obscure simple comparisons of responses to neighbors and strangers. Consequently, we think that the “two hypotheses” paradigm that characterizes individual-level NSRD research (i.e. “familiarity” versus “threat”; Temeles 1994) may be of limited use to guide investigation of variation in conflict between groups. Instead, we need general theoretical models that can predict how individual and group level intergroup responses change as the ecological and social environment changes. Any differential responses to neighbors versus stranger groups over and above those that emerge endogenously from these models would be interesting but, until we have such models, we suspect that NSRD among social groups will usually be attributable to other forms of ecological and social variability.

A challenge for research in this area is the wide gulf that exists between the assumptions of theoretical models of intergroup conflict and the types of social behavior that field and lab studies measure. Most fundamentally, almost all current models of intergroup conflict focus on the evolution of fixed genetic strategies that evolve on an evolutionary time scale, not the evolution of behavioral responses to conflict. To illustrate, it is widely assumed that groups under attack should pull together and become more cohesive, but we are unable to find any formal model to support this prediction in a biological context. What current population genetic and game theoretic models do predict is that an evolutionary history of conflict between groups can, over many generations, favor the spread of alleles for altruism (or reduced conflict effort) within groups, and alleles for hostility between groups (e.g. Choi and Bowles 2007; Lehmann and Feldman 2008; Gavrilets and Fortunato 2014; Barker et al. 2016). Radford and Fawcett (2014) have pointed out this gulf between the assumptions of current theory and the behaviors measured in empirical studies and recommended studying longer-term behavioral responses to try to bridge the gap from the empirical side.

From the theoretical side, there is a need for models that move beyond the assumptions of unitary actors and simultaneous fixed genetic strategies that characterize classic dyadic animal contest theory. One possibility is to build models that specify the sequence or timing of acts involved in conflict (e.g. Thompson et al. 2017), rather than assuming that actors employ simultaneous “sealed bids.” Economic models of “dynamic battles” (Konrad 2009) offer useful exemplars that evolutionary theorists might adapt and explore. Another approach is to solve for bargaining strategies (Binmore 2010), rather than evolutionarily stable fixed strategies. Developing an evolutionary framework to study conflict among heterogeneous and dynamic groups is a technical and conceptual challenge. But, unusually for behavioral ecology, this is a field where theory is lagging behind empirical research, and hence is rich with opportunity. Hopefully, Christensen and Radford’s review will help to motivate and inform new theory to match what empiricists observe and can test.

## FUNDING

F.J.T. and M.A.C. were funded by a grant from the Natural Environment Research Council (NE/N011171/1).
